# Historicity of the ways in which knowledge production impacts practice

**DOI:** 10.1590/1518-8345.0000.2867

**Published:** 2017-11-06

**Authors:** Maria Itayra Coelho de Souza Padilha

**Affiliations:** Graduate Program Volunteer Retired Full Professor at Universidade Federal de Santa Catarina. E-mail: itayra.padilha@ufsc.br

**Figure:**
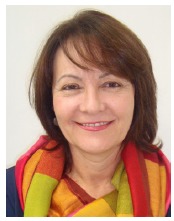

Research is an instrument by which the researcher obtains tools to strengthen the science, but it is also a political act that, as a social practice, contributes to the improvement of the quality of life of the human being. The first issues related to the development of nursing research in Brazil were approached in the official agenda of the 16th Brazilian Congress of Nursing, in 1964, in Salvador/Bahia, entitled, *Nursing and research, nursing care*. Maria Ivete Ribeiro de Oliveira, Dean of the University of Bahia School of Nursing, stated in an keynote lecture the need for a systematized body of scientific knowledge, which, grounded on nursing theories, would be the basis for generalizations, in order to proceed with further investigations, new knowledge and, consequently, the renewal and modernization of professional practice.

The discussions resulted in important decisions. The Brazilian Association of Nursing (Associação Brasileira de Enfermagem - ABEn) was recommended to encourage nursing schools to prepare: their faculty for research, by means of courses and seminars on research methodology; nurses for using research in daily work, producing elements to evaluate the care provided^1^.

The Brazilian nurses dedicated to research can be classified according to the international criteria, namely: the generation of pioneers, which emerged in the 1950s; the generation of those who were self-taught, which emerged in the 1960s; the generation of individual academic development, which appeared in the 1970s; the generation of systematic and collective production, which was established around the 1980s and 1990s; and in 2000s, the generation of scientific production, oriented toward the care settings, through the so-called “good practices”, and practices based on evidence^2^.

Beginning in the 1970s, Brazilian nursing started to develop its own knowledge, based on nursing theories, with the aim of systematization of the care provided by professionals, and stimulating the research of new objects from different qualitative perspectives.

Several factors contributed to the widening of nursing research, many of them coming from ABEn, such as: the Center for Nursing Studies and Research in 1971, which became the repository of Dissertations and Theses defended by Brazilian nurses. This center originated the National Seminar of Nursing Research, in 1979; the development and establishment of the Brazilian Congresses of Nursing, with subjects related to practice needs, and especially, the dissemination and socialization of research results in the periodicals of the health and nursing areas, both nationally and internationally ^(^ ^1^. 

From the first systematized nursing knowledge production until the beginning of the 1980s, the research performed by nurses regarded issues related to the biological component of nursing care, analyses of administrative activities developed by nurses in the institutions, according to functionalist perspectives, standardization and testing of techniques, and studies of normality of biological parameters^3^. The nature of these issues and, fundamentally, the manner of studying them, enabled an advance in the development of a body of nursing knowledge, generating a boost in and a consolidation of this form of research.

From the 1980s, the health area witnessed the emergence of Evidence-Based Medicine (EBM), which stated that scientific findings were more reliable as a basis for clinical decisions than the opinions of authorities, which influenced the other health disciplines of the world^4^. 

Technological advances in the health area and the competitive labor market stimulated nurses to consider specificities of therapeutic care and the identification of nursing’s role in the multiprofessional team. This path motivated the development of specific knowledge, by means of theoretical elaborations, providing a new manner of perceiving the phenomena involved in the care practice. Thus, the need to investigate the objects of nursing with new glasses was disseminated, and qualitative research was used; with that, nursing actions were improved, based on scientific theory. In the 1970s, according to *Maslow’s theory of basic human needs* and the classification of João Mohana, Wanda de Aguiar Horta, proposed the methodology called the “Nursing Process” , which was and still is widely used throughout the country^5^.

The limited use and impact of scientific production related to nursing in the practice setting needs consideration when referring to nursing research in Brazil, as a challenge of the present times. Several hypotheses can be assigned to this, but it is necessary to enable the dissemination and impact of the knowledge produced, as an issue of social responsibility, and considering that, according to ethical and moral reasoning, knowledge must always be shared for the good of humanity. We need to fight for the visibility of nursing research^6^. 

However, it is necessary to understand nursing research as a manner of growth, development and valuing of the profession for society, and not only as an instrument that nurses use in order to obtain knowledge for their daily practice. This means the awakening and understanding of a class that is perceived not only as a work force, but also one that partakes of the full exercise of a profession, making use of the incessant search for knowledge.

In the last 30 years, the scientific knowledge production generated in postgraduate programs, applied to the health needs of the Brazilian population, has enabled an effective articulation of the centers of formation of doctorally- and masters-prepared nurses with the society, in a practice that favored different contexts of health and disease. This new knowledge also renews and modernizes undergraduate education, as well as improve the nursing care in a cycle that establishes and nurtures the work of the profession.

The nursing profession, and that of health in general, followed the evolution of the technological and computational resources for generation and use of information, favoring the improvement of actions aimed at attending the population. In the last decades, initiatives for the development of nursing research, whether individual, collaborative or multi-centered, have multiplied all over the world, with the objective of improving the provision of quality care, patient safety and more effective health policies. Evidence-based practices, clinical research, systematic reviews, convergent-care research, historical studies, phenomenological studies and social representations, have the same goal: to answer questions of professional practice ^(^ ^1^.

I believe that the Brazilian scientific nursing community has expressed concern in promoting, funding and encouraging the development of intervention research, or, in other words, research that provokes changes in care practice, in the face of important health problems. The vacuum that still needs to be filled, in my view, is the strengthening of studies that really aim at the transformation and integration between the scientific community and the care community. Partnerships between schools, health services, and scientists are essential, with a change of roles from the one who produces the knowledge and the one who applies the knowledge that has been produced.
